# Full Sequencing and Genomic Analysis of Three *emm*75 Group A *Streptococcus* Strains Recovered in the Course of an Epidemiological Shift in French Brittany

**DOI:** 10.1128/genomeA.00957-17

**Published:** 2017-09-28

**Authors:** Aude Rochefort, Sarrah Boukthir, Séverine Moullec, Alexandra Meygret, Yahia Adnani, Dominique Lavenier, Ahmad Faili, Samer Kayal

**Affiliations:** a CIC-1414 INSERM, Université Rennes 1, Faculté de Médecine, Team GeRMO, Rennes, France; b CHU Rennes Service de Bactériologie et Hygiène Hospitalière, Rennes, France; c INRIA/IRISA/GenScale, Team Bioinformatique, Campus de Beaulieu, Rennes, France

## Abstract

While the incidence and invasiveness of type *emm*75 group A *Streptococcus* (GAS) infections increased in French Brittany during 2013, we sequenced and analyzed the genomes of three independent strains isolated in 2009, 2012, and 2014, respectively. In this short-term evolution, genomic analysis evidenced mainly the integration of new phages encoding virulence factors.

## GENOME ANNOUNCEMENT

The group A *Streptococcus* (GAS) or *Streptococcus pyogenes* is a Gram-positive human pathogen associated with a broad spectrum of diseases ranging from mild to life-threatening infections (1). The epidemiology varies over time and geographic regions, possibly reflecting the emergence of new virulent clones (2–4). By conducting a systematic survey based on *emm* typing of all GAS isolates in French Brittany, we observed in 2013 a shift in the epidemiological behavior of type *emm*75 GAS infections. During 3 years (2009 to 2012), only 4 infections were recorded, whereas from 2013 to 2014, we documented 27 infections. The multilocus sequence type (5) was determined for all the strains as sequence type 150 (ST150). Anticipating a genetic heterogeneity, we then sequenced the whole genome of three independent *emm*75 strains, here named STAB090229, STAB120304, and STAB14018, isolated from patients with puerperal sepsis (2009), oropharyngeal carriage (2012), and an unexplained bacteremia (2014), respectively.

Whole-genome sequencing was performed with HiSeq 2000 technology (Illumina, Inc., San Diego, CA), and the paired-end libraries were built using the MGX facility of the CNRS in Montpellier, France. For each strain (STAB090229, STAB120304, and STAB14018), a total of 15,201,760, 11,304,212, and 14,127,274 high-quality reads, giving average coverages of 1,253-, 1,068-, and 927-fold, respectively, were assembled using CLC Genomics Workbench v.6 software. The resulting assembly consisted of 25, 25, and 30 contigs oriented on the basis of available sequences of GAS, and 21, 23, and 25 persisting gaps, respectively, were filled as previously described (6, 7). Genome annotations were performed in parallel by using the Rapid Annotations using Subsystems Technology (RAST) server (8) and NCBI-PGAAP (http://ncbi.nlm.nih.gov/genome/annotation_prok). Prophages were identified using the PHAge Search Tool (PHAST) (9).

For each of the three genomes of the sizes 1,846,347, 1,890,354, and 1,890,465 bp, we identified 1,810, 1,868, and 1,804 coding sequences (CDSs), 57, 67, and 67 tRNA genes, and 15, 18, and 18 rRNA genes, respectively. Overall, the main identified genomic differences were focused in 5 intact integrated prophages (ΦSTAB75.1 to ΦSTAB75.5) that varied in G+C percentages from 38.3% to 39.3% and are inserted in intergenic and noncoding regions of the sequenced genomes. The strain STAB090229 integrates 3 prophages (ΦSTAB75.1 to ΦSTAB75.3), and both strains STAB120304 and STAB14018 integrate 4 prophages (ΦSTAB75.1, ΦSTAB75.2, ΦSTAB75.4, and ΦSTAB75.5). The sizes and the genes encoding virulence factors vary for each identified prophage as follows: ΦSTAB75.1 (41.3 kb; streptodorase *sdn*), ΦSTAB75.2 (45.0 kb; SpeL and SpeM exotoxins), ΦSTAB75.3 (55.4 kb; lack of known virulence factors), ΦSTAB75.4 (40.3 kb; SpeC exotoxin), and ΦSTAB75.5 (57.0 kb; SpeK exotoxin). Otherwise, the chromosomal genes encoding SpeG, SmeZ, and NAD glycohydrolase exotoxins and SpeB cysteine protease are found in all sequenced strains.

Until 2013, infections caused by *emm*75 GAS were uncommon in French Brittany. The genetic changes (10), and the acquisition of new virulence factors identified in recovered strains within the same population, demonstrate mechanisms that might explain the short-term shift in epidemiological behavior of type *emm*75 GAS in French Brittany.

### Accession number(s).

The complete whole-genome sequence for each GAS *emm*75 strain is available in GenBank under the accession numbers CP020027 (STAB090229), CP020082 (STAB120304), and CP014542 (STAB14018).
